# Accumulation of arachidonic acid-containing phosphatidylinositol at the outer edge of colorectal cancer

**DOI:** 10.1038/srep29935

**Published:** 2016-07-20

**Authors:** Takanori Hiraide, Koji Ikegami, Takanori Sakaguchi, Yoshifumi Morita, Takahiro Hayasaka, Noritaka Masaki, Michihiko Waki, Eiji Sugiyama, Satoru Shinriki, Makoto Takeda, Yasushi Shibasaki, Shinichiro Miyazaki, Hirotoshi Kikuchi, Hiroaki Okuyama, Masahiro Inoue, Mitsutoshi Setou, Hiroyuki Konno

**Affiliations:** 1 Second Department of Surgery, Hamamatsu University School of Medicine, Higashi-ku, Hamamatsu, Japan; 2 Department of Cellular and Molecular Anatomy, Hamamatsu University School of Medicine, Higashi-ku, Hamamatsu, Japan; 3 International Mass Imaging Center, Hamamatsu University School of Medicine, Higashi-ku, Hamamatsu, Japan; 4 Osaka Medical Center for Cancer and Cardiovascular Diseases, Higashinari-ku, Osaka, Japan; 5 Preeminent Medical Photonics Education & Research Center, Hamamatsu University School of Medicine, Higashi-ku, Hamamatsu, Japan; 6 Department of Anatomy, The University of Hong Kong, Pokfulam, Hong Kong SAR; 7 Division of Neural Systematics, National Institute for Physiological Sciences, Myodaiji, Okazaki, Japan; 8 Riken Center for Molecular Imaging Science, Chuo-ku, Kobe, Japan

## Abstract

Accumulating evidence indicates that cancer cells show specific alterations in phospholipid metabolism that contribute to tumour progression in several types of cancer, including colorectal cancer. Questions still remain as to what lipids characterize the outer edge of cancer tissues and whether those cancer outer edge-specific lipid compositions emerge autonomously in cancer cells. Cancer tissue-originated spheroids (CTOSs) that are composed of pure primary cancer cells have been developed. In this study, we aimed to seek out the cancer cell-autonomous acquisition of cancer outer edge-characterizing lipids in colorectal cancer by analysing phospholipids in CTOSs derived from colorectal cancer patients with matrix-assisted laser desorption/ionization (MALDI)-imaging mass spectrometry (IMS). A signal at *m/z* 885.5 in negative ion mode was detected specifically at the surface regions. The signal was identified as an arachidonic acid (AA)-containing phosphatidylinositol (PI), PI(18:0/20:4), by tandem mass spectrometry analysis. Quantitative analysis revealed that the amount of PI(18:0/20:4) in the surface region of CTOSs was two-fold higher than that in the medial region. Finally, PI(18:0/20:4) was enriched at the cancer cells/stromal interface in colorectal cancer patients. These data imply a possible importance of AA-containing PI for colorectal cancer progression, and suggest cells expressing AA-containing PI as potential targets for anti-cancer therapy.

Colorectal cancer is one of the most deathly tumours worldwide, and great efforts have been made to identify the mechanism of the development and progression, as well as diagnostic biomarkers^1^. Accumulating evidence has indicated that alterations of lipid metabolism contribute to tumour progression in several types of cancer, including colorectal cancer^2^,^3^,^4^. Moreover, lysophosphatidic acids in the stroma have been reported to be involved in cancer progression^5^. *De novo* synthesis of phospholipids and fatty acids is frequently upregulated in cancer cells, even in the early stages of cancer progression^6^,^7^,^8^. This implies that reprogramming of lipid metabolism could be involved in the proliferation, membrane fluidity, and viability of cancer cells by modulating the lipid composition of plasma membrane. The majority of lipidomic analyses reported to date have depended heavily on mass spectrometry (MS)^9^,^10^,^11^. Matrix-assisted laser desorption/ionization (MALDI) imaging mass spectrometry (IMS) is a well established technique nowadays. IMS enables the simultaneous identification and visualization of molecules in biological samples without labelling^12^,^13^,^14^. In particular, IMS has begun to unveil the lipid metabolism of cancer. MALDI-IMS analyses of breast cancer, gastric cancer, hepatocellular carcinoma, and colorectal cancer have offered new insights into the molecular mechanisms underlying carcinogenesis and cancer cell proliferation, migration, and invasion^4^,^15^,^16^,^17^,^18^.

The outer edge of cancer tissue is thought to have unique characteristics, which underlie the property of cancer progression and invasion^19^,^20^,^21^. Growing evidence shows that cancer cells at the cancer outer edge receive stimuli and/or signalling factors from stromal cells. For instance, epithelial-mesenchymal transition (EMT), an important process for tumour invasion, is induced by the interaction between tumour cells at the invasive front and cells in the invaded regions^22^. Despite these findings, it still remains unclear whether the acquisition of characteristics/properties of cancer outer edge occurs in cancer cells autonomously.

Over the past decade, three-dimensional (3D) culture systems that allow cancer cells to proliferate and organize into spheroids have emerged as tractable tools in cancer research^23^. Gene expression patterns in spheroids have been reported to more closely resemble those in real cancer tissues than two-dimensional (2D) cultures^23^. 3D cell culture systems are becoming a powerful tool for bridging the gaps between 2D culture models and *in vivo* studies. However, spheroids originating from a cancer cell line may have a disadvantage of the lacking cell heterogeneity since individual cell lines are homogenous. Recently, Kondo *et al*. developed a state-of-the-art technique to culture spheroids composed of pure primary cancer cells from colorectal cancer, termed cancer tissue-originated spheroids (CTOSs), in which the heterogeneity of parental cancer tissues is preserved^24^. CTOSs retain characteristics of the original tumours, such as *KRAS* and *BRAF* mutations, and have potential to be used in evaluating chemosensitivity of cancer cells derived from individual patients. Despite these advances, spheroids including CTOSs have not been examined in detail.

In this study, we aimed to discover the endogenous lipids characterizing the outer edge of colorectal cancer through analysing the lipid distribution of CTOSs and tumour tissues using MALDI-IMS. We compared phospholipid distribution in CTOSs originated from colorectal cancer patients with that in multicellular tumour spheroids (MCTSs) derived from established cell lines. We provided evidence for an accumulation of an arachidonic acid-containing phosphatidylinositol in the surface of CTOSs and at the outer edge of colorectal cancer cells.

## Results

### Specific accumulation of a molecule with *m/z* 885.5 in the surface region of CTOSs

We first examined the molecular distributions in small regions of CTOSs and MCTSs by analysing the cross-sections at equatorial planes of CTOS and MCTS using IMS analysis at a high spatial resolution. We failed to detect minor lipid species such as lysophospholipids and phosphatidic acids in the high-resolution IMS analysis. Thus, we focused on major lipid species, phosphatidylcholine (PC) and sphingomyelin (SM) in positive ion mode, and phosphatidylglycerol (PG), phosphatidylserine (PS), phosphatidylethanolamine (PE), and phosphatidylinositol (PI) in negative ion mode, which were detected in a narrow range of *m/z* between 700 and 900.

**Figure 1:**
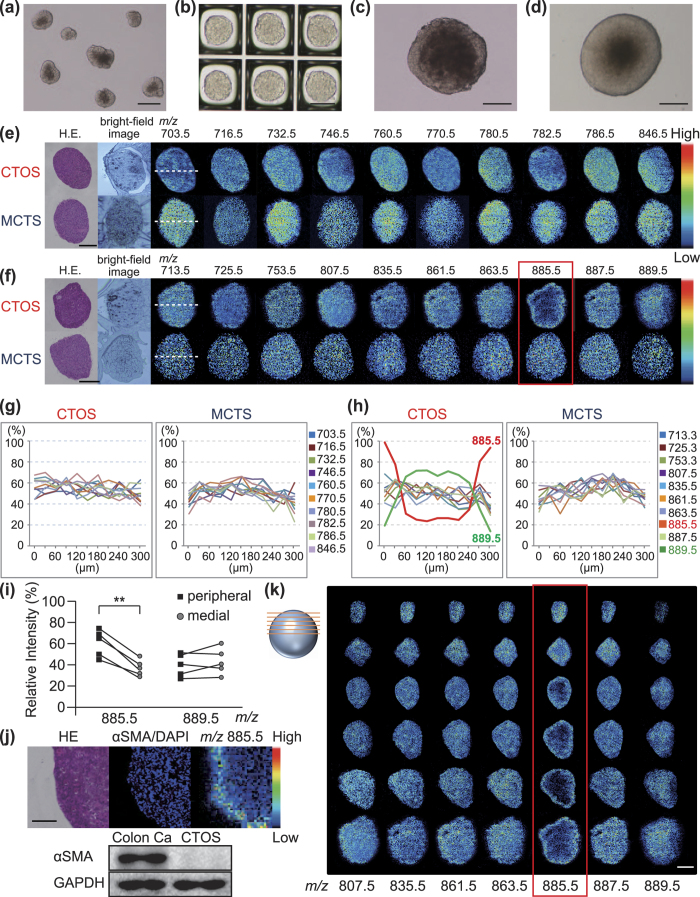
Specific accumulation of a molecule with *m/z* 885.5 in the surface regions of CTOSs. **(a)** CTOSs cultured in suspension medium for 48 hours. **(b)** MCTSs derived from HCT116 cells cultured in Elplasia for 7 days. **(c)** CTOSs embedded and cultured in Cellmatrix Type I-A until its diameter reached 300 μm. **(d)** MCTSs embedded and cultured in the same manner as described in c. Scale bars: 100 μm. **(e,f)** Molecular distributions detected with MALDI-IMS in 10-μm thin sections of investigated CTOS and MCTS in positive (**e**) and negative (**f**) ion modes. Scale bars: 100 μm. Blurred boundary of tissue section in some ion images occurred by low signal-to-noise ratio. **(g,h)** Line scan data showing different distributions of ions along the dashed lines shown in panel (**e**,**f**) in positive (**g**) and negative (**h**) ion modes. **(i)** Comparison of relative signal intensities of *m/z* 885.5 and 889.5 between the peripheral and medial regions of CTOSs. **p < 0.01. **(j)** HE staining, nuclear and α-SMA staining, and MALDI-IMS of CTOS thin serial sections. Left, HE staining; middle, immunohistochemical staining with an anti-α-SMA antibody showing that the tissue was negative for α-SMA, and nuclear staining with DAPI (blue); right, MALDI-IMS of *m/z* 885.5. Scale bar, 50 μm. Western blot analysis of extracts from colorectal cancer tissues and CTOSs. **(k)** MALDI-IMS images of CTOS from 6 slices with 50-μm intervals. Scale bar, 100 μm.

CTOSs and MCTSs with diameters of ~100 μm (Fig. 1a,b) were grown until their diameters reached 300 μm or more (Fig. 1c,d). We analysed CTOSs and MCTSs, the diameter of which was 300 μm or more. A large number of signals were detected in both positive (Supplementary Fig. S1a) and negative (Supplementary Fig. S1b) ion modes. In positive ion mode, no molecule showed a distribution in CTOSs that is distinctly different from that in MCTSs (Fig. 1e,g). In negative ion mode, in contrast, some molecular species accumulated in restricted regions of CTOS samples. A molecule with *m/z* 885.5 was more strongly detected in the periphery of CTOS sections than in the medial regions (Fig. 1f,h). In contrast, a molecule with *m/z* 889.5 was detected preferentially in the medial regions of CTOSs than in the peripheral regions (Fig. 1f,h). Other molecular species were detected more uniformly than these two molecules (Fig. 1f,h). All the analysed molecules, including the two molecules with *m/z* 885.5 and 889.5, were uniformly detected in MCTS sections (Fig. 1f,h).

We further performed an unsupervised multivariate analysis, principal component analysis (PCA) of pixel-to-pixel comparison, on imaging mass spectrometry, to seek out other minor ion species that segregate the periphery and medial region of CTOS. PC3 successfully segregated the periphery and medial region of CTOS (Supplementary Fig. S2a). Ion of *m/z* 885.5 and its isotopic peak *m/z* 886.5 were detected as most affecting factors of PC3, showing the highest loading scores (Supplementary Fig. S2a). We failed to find other molecules that accumulated at the periphery of CTOS. The PCA did not detect subpopulations in MCTS section (Supplementary Fig. S2b).

Focusing on negative ion mode, we compared and statistically analysed signal intensities of the molecules in the peripheral and medial regions of five CTOSs prepared from different patients. In this analysis, the medial region of a CTOS was defined as 80% of the CTOS radius. Only the molecule with *m/z* 885.5 showed a statistically significant difference in signal intensities between the peripheral and medial regions (*P* = 0.001) (Fig. 1i and Supplementary Fig. S3). The molecule with *m/z* 889.5 showed a trend of accumulating in the medial region of CTOSs although the difference is not statistically significant (*P* = 0.223) (Fig. 1i and Supplementary Fig. S3). The higher magnification required to inspect the CTOS thin sections meant that the peripheral and medial regions were hard to be distinguished, as evident from the cell density as well as tissue density (Fig. 1j). Furthermore, the CTOSs were negative for α-SMA, a marker for activated fibroblasts, whereas α-SMA was detected in the colorectal cancer tissues (Fig. 1j).

We analysed six serial thin sections of a CTOS hemisphere prepared with an interval of 50 μm to examine whether the molecule with *m/z* 885.5 was abundant in the surface of the CTOS. The signal at *m/z* 885.5 was uniformly detected in the first section prepared from the surface of CTOS (Fig. 1k, first row). In the second section, which was 50-μm deeper than the first one, the signal at *m/z* 885.5 was slightly weaker in the medial region than in the peripheral region (Fig. 1k, second row). Stronger signals at *m/z* 885.5 were detected in the circumferences of all other sections (third, fourth, fifth, and last sections) than in the medial regions (Fig. 1k, third to sixth rows), while the signal at *m/z* 889.5 was weakly detected in the first section (Fig. 1k, first row). These results indicate that the molecule with *m/z* 885.5 was selectively accumulated at the surface of the investigated CTOS.

### Identification of molecules with *m/z* 885.5 and *m/z* 889.5 as PI(18:0/20:4) and PI(18:0/20:2), respectively

**Figure 2:**
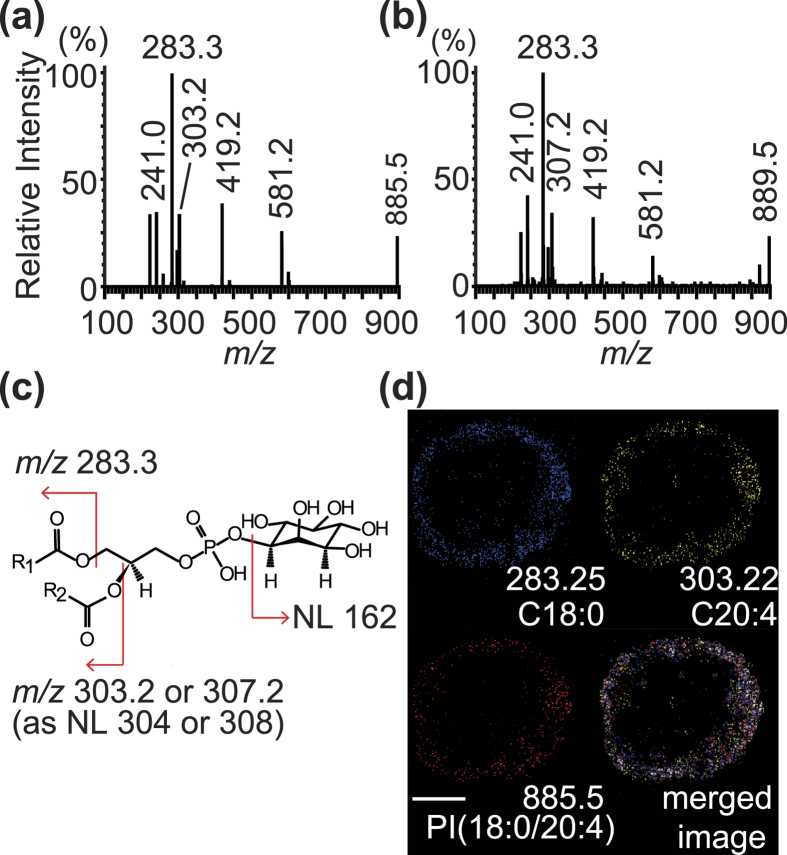
Identification of the molecules with *m/z* 885.5 and *m/z* 889.5 as PI(18:0/20:4) and PI(18:0/20:2), respectively. **(a,b)** Tandem mass spectra of product ions derived from *m/z* 885.5 (**a**) and 889.5 (**b**). **(c)** Cleavage patterns of PI(18:0/20:2) and PI(18:0/20:4). The difference in R2 produces different product ions, one with *m/z* 303.2 and the other 307.2. **(d)** Images derived from MS/MS imaging for the molecule with *m/z* 885.5. Scale bar, 100 μm.

We next identified the molecules with *m/z* 885.5 and *m/z* 889.5 by tandem mass spectrometry (MS/MS) analysis. In the MS/MS analysis, the precursor ion with *m/z* 885.5 was fragmented into product ions with *m*/*z* 581.2, 419.2, 303.2, 283.3, and 241.0 (Fig. 2a). The product ion with *m*/*z* 581.2 corresponded to a molecule generated by a neutral loss of arachidonic acid (AA; 304 Da, C20:4). The product ion with *m*/*z* 419.2 was identified as a molecule resulting from a neutral loss of inositol (162 Da; C_6_H_10_O_5_) and AA. The peak at *m*/*z* 303.2 corresponded to a deprotonated form of AA whereas the peak at *m/z* 283.3 corresponded to stearic acid (284 Da, C18:0) with a loss of a H^+ ^ion. The peak at *m*/*z* 241.0 was phosphoryl inositol (260 Da, C_6_H_12_O_5_PO_4_) losing a H_2_O molecule. These data indicated the molecule with *m*/*z* 885.5 as phosphatidylinositol (PI) (18:0/20:4). A precursor ion with *m*/*z* 889.5 generated product ions with *m*/*z* 581.2, 419.2, 307.2, 283.3, and 241.0 (Fig. 2b). The peak at *m*/*z* 307.2 corresponded to eicosadienoic acid (C20:2) lacking a H^+^ ion. The molecule with *m/z* 889.5 was indicated as PI(18:0/20:2). The fragmentation patterns of the molecules with *m/z* 885.5 and 889.5 were found to match those of PI(18:0/20:4) and PI(18:0/20:2), respectively, in the Human Metabolome Database (HMDB; http://www.hmdb.ca/) (Fig. 2c). We further performed tandem mass spectrometry (MS/MS) imaging to confirm our finding. The precursor ion with *m/z* 885.5 and product ions with *m/z* 283.3 and 303.2 were strongly detected in the periphery of the investigated CTOS (Fig. 2d).

### Preferential accumulation of PI(18:0/20:4) in the surface regions of CTOSs

**Figure 3:**
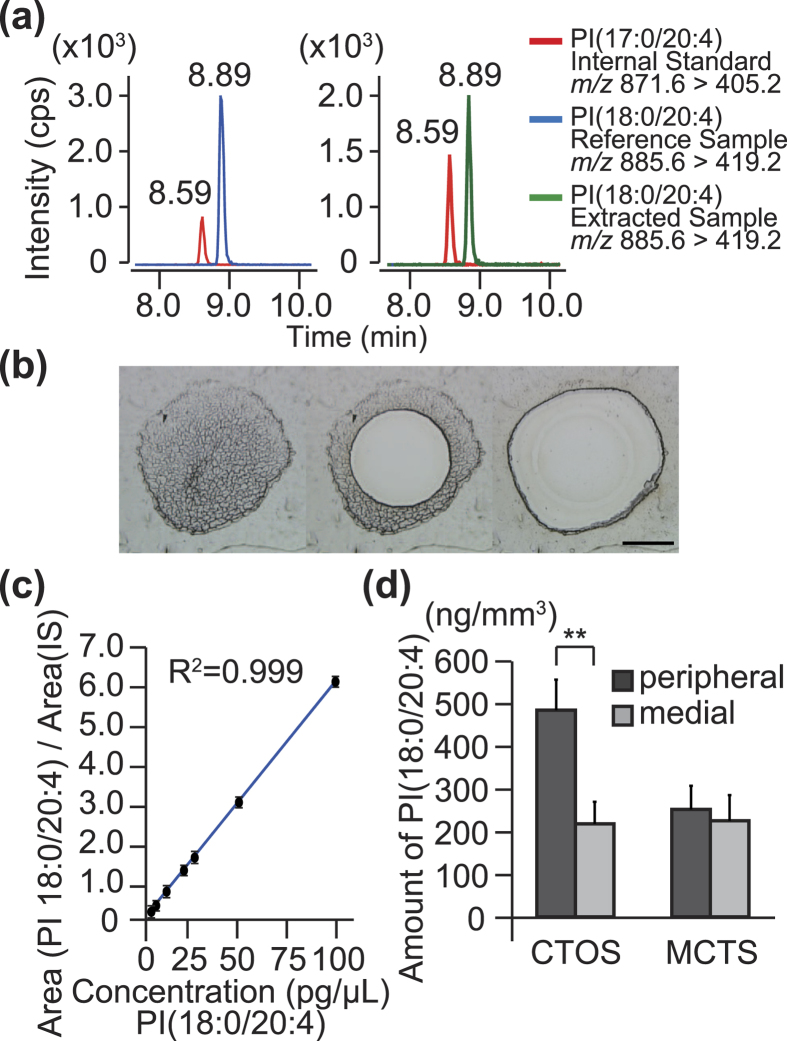
A two-fold higher amount of PI(18:0/20:4) in the surface regions than in the medial regions of investigated CTOSs. **(a)** Chromatogram of reverse phase-HPLC-ESI-MS/MS. The red peaks were PI(17:0/20:4) loaded as an internal standard (IS). The blue peak was pure PI(18:0/20:4) loaded as a reference sample. The green peak was detected in the lipid extract of CTOSs. The *m/z* values of precursor ions and product ions were provided. **(b**) A micro-dissected CTOS thin section. The medial region (defined as the area covered by the 80% length of the radius measured from the centre of a spheroid) and the peripheral region (the area covered by the remaining distal 20%) were separately collected. Scale bar, 100 μm. **(c)** A calibration curve for PI(18:0/20:4). **(d)** Quantification of PI(18:0/20:4) in the peripheral and medial regions of MCTSs and CTOSs. Values are mean ± SD, n = 5. **P < 0.01.

We further examined whether the molecule with *m/z* 885.5 was PI(18:0/20:4) by liquid chromatography (LC)-MS/MS analysis. To this end, we used pure PI(18:0/20:4) as a reference and pure PI(17:0/20:4) as an internal standard (IS). The retention times of pure PI(18:0/20:4) and PI(17:0/20:4) were 8.89 and 8.59 min, respectively (Fig. 3a, left). The retention time of the molecule with *m/z* 885.5 extracted from investigated CTOSs was also 8.89 min (Fig. 3a, right). Moreover, both pure PI(18:0/20:4) and the investigated molecule produced a major product ion with *m/z* 419.2 in the MS/MS analysis (Fig. 3a). These results verified the molecule with *m/z* 885.5 as PI(18:0/20:4).

We next measured the amount of PI(18:0/20:4) extracted separately from the peripheral and medial regions of CTOSs using laser capture microdissection (LCM) by LC-MS/MS analysis (Fig. 3b). The calibration curves were highly linear in the range between 5 pg/μL and 100 pg/μL (R^2^ = 0.999) (Fig. 3c). Recovery efficiency of PI(18:0/20:4) in the small and thin sections of spheroids was about 40%: 243.5 ± 54.1 ng/mm^3^ of LCM-dissected section versus 576.9 ± 82.7 ng/mm^3^ of whole tissues (Supplementary Fig. S4). The amount of PI(18:0/20:4) was ~2-fold higher in the peripheral regions (494.1 ± 73.0 ng/mm^3^) than in the medial regions of investigated CTOSs (222.3 ± 52.7 ng/mm^3^; *P* = 0.009) (Fig. 3d). In contrast, there was no statistically significant difference in the lipid contents between medial and peripheral regions of MCTSs derived from HCT116 cells (230.0 ± 61.0 ng/mm^3^ versus 257.1 ± 56.1 ng/mm^3^) (Fig. 3d).

### Accumulation of PI(18:0/20:4) at the outer edge of cancer cells in colorectal cancer tissues

**Figure 4:**
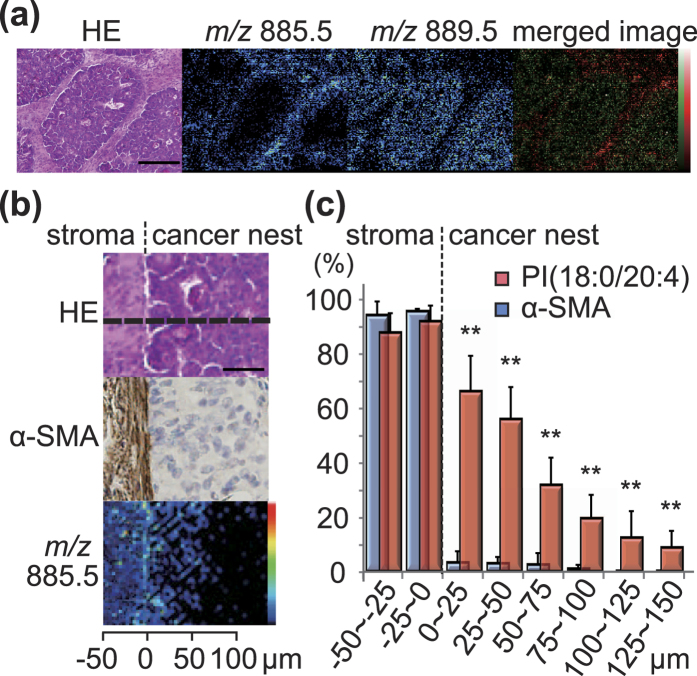
Accumulation of PI(18:0/20:4) at the outer edge of cancer cells in colorectal cancer tissues. (**a)** HE staining and MALDI-IMS of serial thin sections of a colorectal cancer tissue sample. Scale bar, 200 μm. The most right panel is a merged image of MALDI-IMS of *m/z* 885.5 (red) and 889.5 (green). **(b)** Higher magnification of serial thin sections of the colorectal cancer tissue sample. Upper panel, HE staining; middle panel, immunohistochemical staining of α-SMA; lower panel, MALDI-IMS of *m/z* 885.5. **(c)** Quantified signal intensities of PI(18:0/20:4) (*m/z* 885.5) as detected on the region marked with the dashed line as shown in panel b. The data are mean ± SD, n = 20. **P < 0.01.

Finally, we investigated the possible clinical relevance of the increased accumulation of PI(18:0/20:4) in the periphery of CTOSs by analysing colorectal cancer tissues with IMS. The cancer cells of colorectal cancer were readily distinguished from cancer-surrounding stromal tissues by HE staining (Fig. 4a and Supplementary Fig. S5a). PI(18:0/20:4) (*m/z* 885.5) was strongly detected in the stroma, but not in the medial regions of cancer where PI(18:0/20:2) (*m/z* 889.5) was detected (Fig. 4a and Supplementary Fig. S5b,c,d). Interestingly, highly-magnified microscopy clearly showed that PI(18:0/20:4) was strongly detected at the outer edge of cancer cells that contacted stromal tissues (Fig. 4b). The outer edge of cancer cells positive for PI(18:0/20:4) were completely negative for α-SMA, a specific marker for stromal cells (Fig. 4b). This rules out the possibility of the presence of fibroblasts in the outer edge of the cancer cells. Quantification of signal intensities of α-SMA and PI(18:0/20:4) further verified that PI(18:0/20:4) accumulated at the stroma/ tumour interface of growing colorectal tumours (Fig. 4c). Other PIs, including PI(16:0/16:1), PI(16:0/18:1), PI(18:0/18:2), PI(18:0/18:1), and PI(18:0/20:3), which were detected in CTOSs, were also detected in the cancer cells (Supplementary Fig. S5e).

## Discussion

In this study, we attempted to reveal phospholipids that characterized the outer edge of colorectal cancer cells, and found an AA-containing PI, PI(18:0/20:4) as a cancer outer edge-characterizing phospholipid in colorectal tumours. We first noticed a unique distribution of an AA-containing PI, PI(18:0/20:4), in CTOSs originating from colorectal cancer patients, but not in MCTSs derived from homogeneous cell lines. We then found a similar distribution of PI(18:0/20:4) in colorectal cancer tissues. Strikingly, as well as being predominantly detected at the surface of CTOSs, PI(18:0/20:4) specifically accumulated at the outer edge of colorectal cancer cells that are in contact with stromal tissues. Consistent with a previous study^25^, PI(18:0/20:4) was predominantly detected in tumour stroma. One of the simplest possible explanations is that the outer edge of a cancer cell is a mixture of stromal fibroblasts and tumour cells. Our data clearly exclude this possibility, since the outer edge of investigated cancer cells were negative for α-SMA, a specific marker of fibroblasts. Furthermore, our data suggest that the specific accumulation of PI(18:0/20:4) at the outer edge of a cancer cells occurs independently of stromal cells, since CTOSs contain neither stromal cells nor stromal factors. Given the lipid recovery efficiency of 40% with LCM and that CTOSs with diameters of 100 μm contain ~100 cells^24^, the estimated amount of PI(18:0/20:4) in the surface of CTOSs is around 7 fmoles/cell, which seems to be in the physiological range (~10 fmoles/cell in liver and brain)^26^.

What biological roles does PI(18:0/20:4) have at the outer edge of colorectal cancer? A possible answer is that PI(18:0/20:4) accounts for the migration of cancer cells at the outer edge. This explanation fits well with a finding that AA incorporation into PI is required for neuronal cell migration^27^. Molecular mechanisms underlying such a function can be explained according to the molecular properties of PI(18:0/20:4), although direct evidence remains to be provided. PI(18:0/20:4) is known to be the most abundant PI in normal tissues^28^. Some of the enzymes that contribute to PI cycle prefer PI(18:0/20:4) as substrates^29^. Importantly, PI(18:0/20:4) is preferentially converted to phosphatidylinositol polyphosphates (PIPs), which play key roles in a wide range of cellular processes, including cell migration, invasion, and proliferation^29^. In particular, phosphatidylinositol 3,4,5-trisphosphate (PIP3) converted from PI(18:0/20:4) by PI3 kinase triggers the activation of the Akt pathway, and subsequently activates various tumour-promoting processes^30^,^31^. Notably, it is well known that cancer cells at the invasive front undergo EMT^22^. The selective accumulation of PI(18:0/20:4), not only at the outer edge of cancer cells but also in the surface of CTOSs, could account for their aggressive activities, including enhanced proliferation and invasion. Indeed, Kondo *et al*. have reported that proliferating cells are concentrated predominantly in the surface regions of CTOSs^24^.

Another question is what mechanisms underlie the accumulation of PI(18:0/20:4) at the outer edge of cancer cells and at the periphery of CTOSs. PI accounts for 10% of the phospholipids constituting plasma membrane. The major phospholipids that constitute plasma membrane are PCs, which account for 50% of the phospholipids. Interestingly, other AA-containing PCs, such as PC(16:0/20:4) + K^+^ (*m/z* 820.5) and PC(18:1/20:4) + K^+^ (*m/z* 846.5) were not detected in the surface regions of CTOSs. These data suggest that the incorporation of AA into PI is specifically increased. One possible answer is the difference in the concentration of available oxygen. Importantly, oxygen supply can promote the incorporation of AA into PI^32^,^33^. The surface regions of cancer cells and CTOSs, where PI(18:0/20:4) was strongly detected, are more efficiently supplied with oxygen than their medial regions^34^. This could result in the preferential uptake of AA into PI in the surface regions. Another possibility is that serum- or plasma-carried factors stimulate AA incorporation into PI in the surface regions of cancer cells and CTOSs. A candidate for such factors is platelet-derived growth factor (PDGF). PDGF has been reported to stimulate AA incorporation into PI^35^,^36^. In contrast, we failed to observe any trend in the distribution of PI(18:0/20:4) in MCTSs, although the supply of oxygen and serum-carried factors was similar to that in CTOSs. A simple but revolutionary explanation for this discrepancy is the heterogeneity of cells composing CTOSs and cancer cells. Cells of established cell lines are, by contrast, highly homogeneous due to cloning steps in many cases^37^. Our data suggest that the heterogeneous cancer cells in CTOSs and cancer cells in colorectal cancer harbour a subpopulation of cells with a high potency of accumulating PI(18:0/20:4) in putative oxygen- and/or PDGF-dependent manners, although additional studies are needed to confirm this proposition. Indeed, our pixel-to-pixel PCA on IMS images detected the heterogeneity of CTOSs successfully, but not that of MCTSs.

PI(18:0/20:4) is subjected to the Lands cycle. In the cycle, PI(18:0/20:4) is degraded into AA and lysophosphatidylinositol (LPI) by phospholipase A2 enzymes (PLA2s), and regenerated through the incorporation of AA into the *sn2* position of LPI by lysophosphatidylinositol acyl-transferase 1 (LPIAT1, also known as MBOAT7)^26^,^27^,^38^. Both the enhancement of LPIAT1-mediated regeneration and the suppression of PLA2-mediated degradation can, thus, cause the accumulation of PI(18:0/20:4). LPIAT1 is highly specific for the incorporation of AA into LPI, whereas PLA2s degrade a broad variety of phospholipids, including PCs, to release AA. Our data show that other AA-containing PCs were not detected in the surface regions of CTOSs and colorectal cancer cells, suggesting that PLA2-mediated degradation of AA-containing phospholipids could be active. Hence, an enhanced LPIAT1 activity seems to underlie the specific accumulation of PI(18:0/20:4) in the surface regions, overwhelming PLA2 activity. PI(18:0/20:4) can also be degraded by phospholipase C enzymes (PLCs) after it is converted to phosphatidylinositol 4,5-bisphosphate (PIP2)^30^. However, the contribution of this degradation pathway may not be significant, since only a small portion of PIs are converted to PIP2^30^. These molecular insights propose LPIAT1 as a prospective target in blocking colorectal cancer progression.

In conclusion, our study demonstrates for the first time the unique accumulation of an AA-containing PI, PI(18:0/20:4), at the outer edge of colorectal cancer cells. These data imply the possible importance of AA-containing PIs for the progression of colorectal cancer, including invasion, and suggest PI(18:0/20:4) as a possible biomarker for metastatic stage of colorectal cancer and LPIAT1 as a potential therapeutic target for colorectal cancer.

## Materials and Methods

### Ethics statement

All the experiments in this study were specifically approved by the Ethics Committee at the Hamamatsu University School of Medicine and the methods were carried out in accordance with the approved guidelines. Informed consent was obtained in a written form from each patient before performing each operation. The subjects consented to cooperate after they were informed that they would not incur any disadvantage, that they could resign from the study, that the researchers were obliged to protect their privileged information, and that their identities would not be revealed. Totally, 20 patients participated in this work at the Hamamatsu University School of Medicine.

### Tumour Tissue and CTOS preparation

Colorectal cancer tissues which were surgically obtained were flash-frozen in liquid nitrogen and stored at −80 °C for histological experiments. Cancer regions were obtained for CTOS preparation. CTOSs were prepared as previously described^24^. CTOSs were embedded and cultured in Cellmatrix type I-A (Nitta Gelatin) droplets with stem cell medium until spheroids grew up to a diameter of 300 μm; 11 of the 20 samples (55%) reached a diameter of ≥300 μm (Table 1). CTOSs were released from the Cellmatrix by incubation with 0.2 mg/mL collagenase type IV (Worthington) and cultured in suspension in stem cell medium for one day.

### Preparation of MCTSs

HCT116 and DLD-1 colon carcinoma cell lines were obtained from American Type Culture Collection (Manassas, VA) and were authenticated via short tandem repeat fingerprinting by the Japanese Collection of Research Bioresources Cell Bank. The micro-space cell culture plate, Elpasia was kindly gifted by Kurarey Co., Ltd., Tsukuba, Japan. The cells were cultured on Elplasia coated with poly 2-hydroxyethyl methacrylate (p-HEMA) solution in stem cell medium according to the manufacturer’s instructions. After incubation for 7 days, MCTSs of about 100 to 150 μm in diameter were detached from the plates by gentle pipetting. MCTSs were also embedded and cultured in Cellmatrix type I-A droplets with stem cell medium until spheroids grew up to a diameter of 300 μm. MCTSs were released using the same method as described for CTOSs and cultured in suspension in stem cell medium for one day.

### Sample preparation for MALDI-IMS analysis

CTOSs and MCTSs were washed with Hank’s Balanced Salt Solution without calcium and magnesium (Gibco). CTOSs and MCTSs were embedded in gelatine solution according to a previous report^39^. After solidification, the pellets were immediately frozen at −80 °C for storage. For IMS measurements, the pellets were sectioned to a thickness of 10 μm using a Leica CM1950 cryostat (Leica Microsystems GmbH) at −25 °C. Only samples that were not crushed during sectioning were used: 5 CTOSs out of 11 samples were successfully prepared. The sections were thaw-mounted onto indium-tin-oxide (ITO)-coated glass slides (Bruker Daltonics). Each ITO slide that held up to 8–12 spheroid sections was dried in a desiccator at room temperature prior to matrix deposition. 2′,5′-dihydroxyacetophenone (DHAP) (Tokyo Chemical Industry CO., LTD.) was used as the matrix and deposited on the sections using a matrix sublimation instrument (Shimadzu Corporation). Matrix sublimation was performed as previously described^40^. Colorectal cancer tissues were also sliced to a thickness of 10 μm using the Leica cryostat and matrix sublimation was performed. The condition of matrix deposition was checked by both megascopic and microscopic observations, and samples that were uniformly coated by matrix were analysed with IMS. Serial sections of CTOSs, MCTSs, and colorectal cancer tissues were collected onto MAS-coated glass slides and subjected to HE staining and immunohistochemical staining. Photomicrographs of stained sections were acquired using a Keyence BZ-9000 fluorescence microscope (Keyence).

### Atmospheric pressure MALDI-IMS and tandem mass spectrometry (MS/MS) analyses

MALDI-IMS analysis was performed using a high-resolution imaging mass spectrometer (iMScope prototype; Shimadzu) equipped with a 355-nm Nd:YAG laser^41^. The measurement regions were determined using a microscope equipped inside the instrument. The sections of CTOSs, MCTSs, and colorectal cancer tissues were observed with 20x magnification lens. Both the diameters of focused laser spot and raster scan pitch were set to 5 μm. At each measurement point, signals were accumulated by irradiating 100 laser shots with a repetitive frequency of 1,000 Hz to acquire mass spectra in the mass-to-charge ratio (*m/z*) range of 700–900 in both positive and negative ion modes. Distributions of molecules were analysed by reconstructing ion images using signal intensities displayed in the mass spectra. We also performed MS/MS imaging using the same instrument to identify lipid species and their fatty acid composition from product ion spectra.

### Immunohistochemistry and western blotting

Immunohistochemistry was performed as previously described^42^. Anti-α-smooth muscle actin (α-SMA) antibody (1:100, Dako) was used as a primary antibody. Western blotting was carried out as previously described^43^. Anti-glyceraldehyde 3-phosphate dehydrogenase (GAPDH) antibody (Merck Millipore) and anti-α-SMA were used as primary antibodies at a dilution of 1:1000.

### Laser capture microdissection (LCM) and lipid extraction

To extract lipids from CTOSs, gelatine-embedded spheroid pellets were sliced at a thickness of 16 μm using a Leica CM1950 cryostat at −25 °C and placed onto polyethylene naphthalate (PEN) slides (11505158, Leica). The tissue sections were then immediately microdissected by means of the Leica LMD-6000 laser dissection microscope. Small tissue pieces of 1.0 mm^2^ were collected separately from the medial and peripheral regions of CTOS samples. The medial region was defined as the area covered by 80% of the length of the radius measured from the centre of a spheroid. The 80% of the radius determined as follows: We determined the maximum and minimum diameter of the CTOS sections. We measured two points from maximum and minimum diameter which were 80% of the length of the radius of the spheroid. Then, CTOS sections were cut in ellipse mode. The small tissues pieces were collected into plastic tubes containing 50 μL of ultrapure water (Wako). PI(17:0/20:4) (Avanti Polar Lipids) was added to the samples at 1 pmol (8.7 μL of 0.115 μM) as an internal standard. Phospholipids were extracted by the Bligh and Dyer method^44^. To improve recovery efficiency, the commonly utilized HCl-acidified extraction method was performed^45^. Concentrated phospholipid extracts were dissolved in 100 μL of methanol.

To evaluate the yield of lipids from the small and thin portions of spheroids, gelatine-embedded MCTSs were used. For LCM-based lipid extraction, whole MCTSs were sliced at a thickness of 16 μm, and the thin sections were placed onto PEN slides. Lipids were extracted separately from the peripheral and medial regions of the thin sections. As a reference, lipids were extracted from a whole MCTS (300 μm in diameter) by homogenizing the spheroid in 50 μL of pure water without slicing or microdissection.

### LC-MS/MS analysis

Lipids were separated with the ACQUITY Ultra Performance LC (UPLC) system (Waters). Five μL of lipid extracts were loaded onto ACQUITY UPLC BEH C18 column (1.7 μm particle size, 2.1 × 50 mm; Waters) maintained at 40 °C. The column was connected to an ACQUITY UPLC VanGuard BEH C18 guard column (2.1 × 5 mm; Waters). The samples were eluted with ammonium acetate (Wako) linearly decreased from 4 mM to 0 mM and methanol (Wako) linearly increased from 80% to 100% at 200 μL/min for 10 min, followed by an additional elution with 100% methanol for 2 min.

MS/MS analyses were performed in negative ion mode using a quadrupole-linear ion trap hybrid mass spectrometer (4000 QTRAP, Applied Biosystems/MDX Sciex) equipped with an interface of electrospray source ionization (ESI). The ion spray voltage and declustering potential were −4500 V and −35 V, respectively. Collision energy for both PI(17:0/20:4) and PI(18:0/20:4) were set at −56 V. Deprotonated ions ([M-H]^−^) were chosen as precursor ions for the MS/MS analysis. In the selected reaction monitoring (SRM) of PI(17:0/20:4), *m*/*z* 871.6 was monitored in the first quadrupole (Q1), and *m/z* 405.2 was monitored in the third quadrupole (Q3). For the SRM of PI(18:0/20:4), *m*/*z* 885.6 and 419.2 were monitored in Q1 and Q3, respectively. The calibration curve for PI(18:0/20:4) was generated with different concentrations (1, 2.5, 5, 10, 12.5, 25, 50, 75, and 100 pg/μL) of PI(18:0/20:4) (Avanti Polar Lipids) solubilized in methanol. PI(17:0/20:4) at 10 nM was used as an internal standard. Data were analysed by the Analyst software (Applied Biosystems/MDX Sciex).

### Data analysis

For the line scan analyses, mass images were exported as greyscale images, and signal intensities (SI) were line-scanned on a line of 5 pixels in width from the peripheral to the centre region by using the Image J software^46^. Ions detected in both CTOSs and MCTSs in all five independent experiments with signal-to-noise (S/N) ratio three or more were selected.

A pixel-to-pixel principal component analysis (PCA) was performed using the Mass Microscope System software (Imaging MS Solution, Shimadzu). Top 100 peaks whose the signal-to-noise (S/N) ratio of signals are larger than two were selected, and subjected to PCA with pareto scaling and varimax rotation. Primary component score images and factor loading spectra were provided.

Statistical analyses (paired *t*-test for Fig. 1 and Supplementary Fig. S3; Mann-Whitney U-test for Figs 3 and 4, and Supplementary Fig. S4) were performed using the IBM SPSS Statistics software (version 21, IBM). A *P* value of <0.05 was considered significant.

## Additional Information

**How to cite this article**: Hiraide, T. *et al*. Accumulation of arachidonic acid-containing phosphatidylinositol at the outer edge of colorectal cancer. *Sci. Rep.* **6**, 29935; doi: 10.1038/srep29935 (2016).

## Supplementary Material

Supplementary Information

## Figures and Tables

**Table 1:** Patient characteristics and CTOS establishment.

Patient characteristics	Quantity	Note
Sex		
Male	8	
Female	12	
Age (years)		
＜60	7	
≧60	13	
Mean ± SD	65.2 ± 14.3	
Tumour location		
Cecum	2	
Ascending colon	2	
Transverse colon	1	
Descending colon	1	
Sigmoid colon	5	
Rectum	8	
Proctodeum	1	
Stage (UICC)		
I	1	
II	8	
IIIa, b	10	
IV	1	Lung
Histopathological grading		
Well differentiated	7	
Moderately differentiated	11	
Poorly differentiated	2	
CTOS	%	Note
Formation	100	20 out of 20
Growth (>300 μm)	55	11 out of 20
