# Supplementary material for: Accumulation of arachidonic acid-containing phosphatidylinositol at the outer edge of colorectal cancer
Source: Sci Rep. 2016 Jul 20;6:29935. doi: 10.1038/srep29935 (PMC4951683; doi:10.1038/srep29935)
Supplement: Supplementary Information [file srep29935-s1.pdf]

Supplementary Information

Title:

Accumulation of arachidonic acid-containing phosphatidylinositol at the outer edge of colorectal cancer

Authors:

Takanori Hiraide, Koji Ikegami, Takanori Sakaguchi, Yoshifumi Morita, Takahiro Hayasaka, Noritaka Masaki, Michihiko Waki, Eiji Sugiyama, Satoru Shinriki, Makoto Takeda, Yasushi Shibasaki, Shinichiro Miyazaki, Hirotoshi Kikuchi, Hiroaki Okuyama, Masahiro Inoue, Mitsutoshi Setou*, and Hiroyuki Konno

Corresponding author: Mitsutoshi Setou

International Mass Imaging Center, and Preeminent Medical Photonics Education & Research Center, and Department of Cellular and Molecular Anatomy, Hamamatsu University School of Medicine, 1-20-1 Handayama, Higashi-ku, Hamamatsu 431-3192, Japan.

Phone: +81-53-434-5282; Fax: +81-53-435-2473, E-mail: setou@hama-med.ac.jp

Supplementary Figure

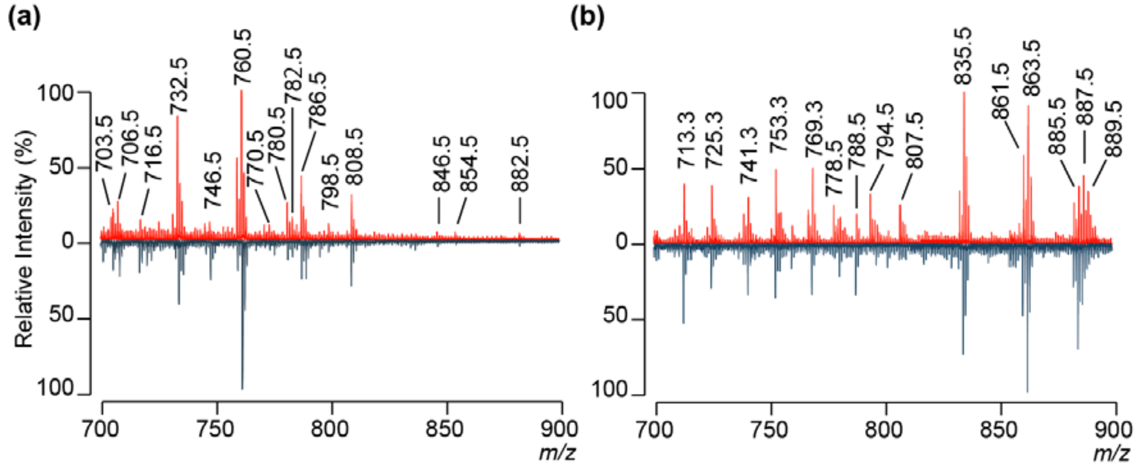

Supplementary Fig. S1. Average mass spectra of MALDI-IMS on CTOS and MCTS sections. (a) and **(b)** Mass spectra acquired in positive (a) and negative (b) ion modes. Upper spectra (red), CTOS; lower spectra (blue), MCTS.

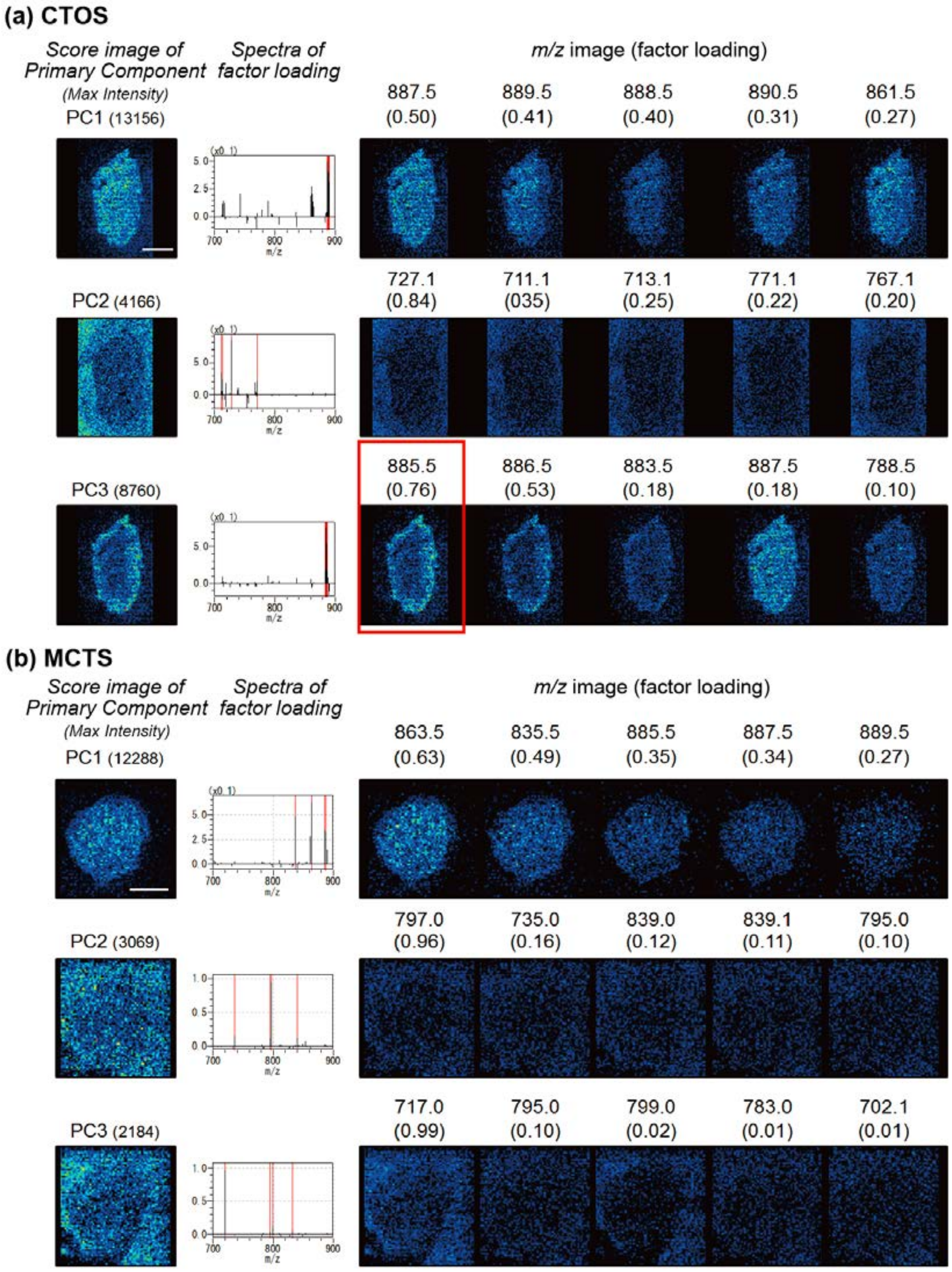

Supplementary Fig. S2. Principle component analysis acquired from IMS of CTOS and MCTS section. (a, b) The top three principal components and top five loadings were shown. Scale bars, 100 µm. (a) PC3 segregated the peripheral and medial regions of CTOS. *m/z* 885.5 had the highest loading score of PC3. PC1 and PC2 distinguished the tissue section and glass surface. (b) PCA failed to detect specific regions in MCTS section.

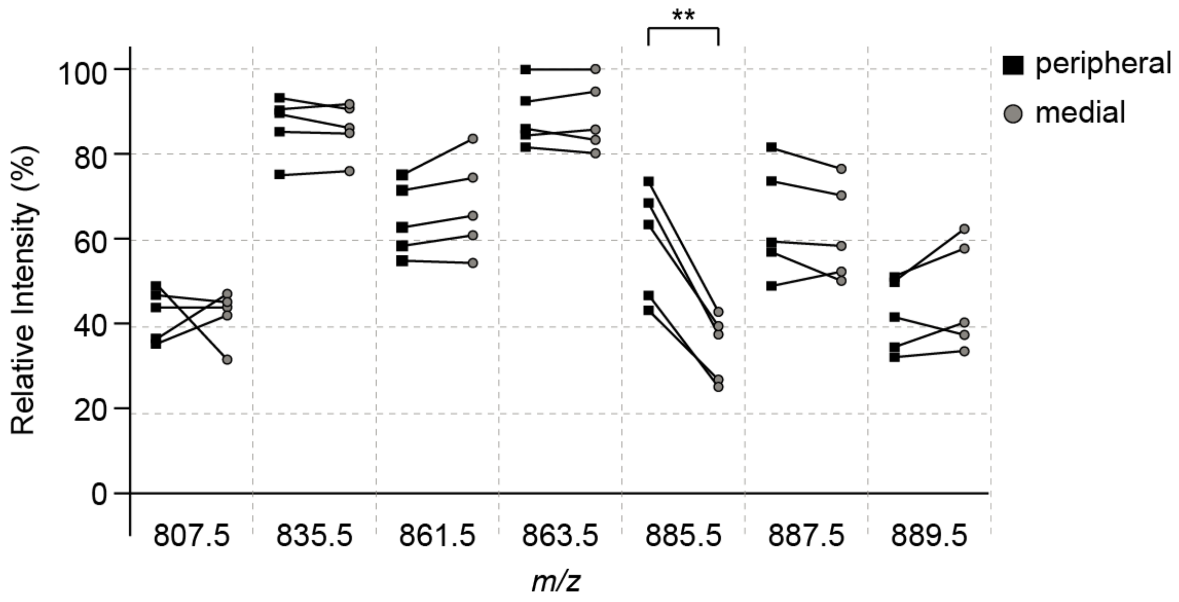

Supplementary Fig. S3. Mean relative signal intensities of seven molecules with *m/z* 807.5, 835.5, 861.5, 863.5, 885.5, 887.5, and 889.5 at the peripheral and medial regions of CTOSs. Signal intensities of the indicated molecules in the peripheral regions were compared with those in the medial regions of five CTOSs prepared from independent patients. **, $P < 0.01$.

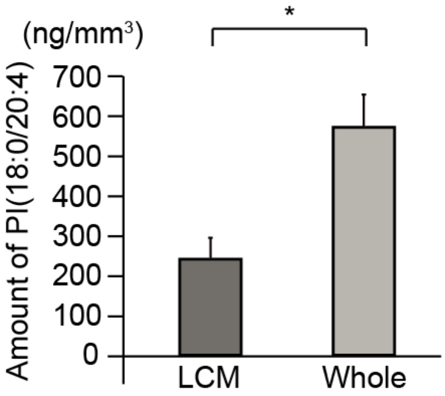

Supplementary Fig. S4. Evaluation of extraction efficiency of PI(18:0/20:4) with LCM. Recovery efficiency of PI(18:0/20:4) was assessed by LCM-based lipid quantification for the small and thin sections of MCTSs. Values are mean ± SD, n = 5. *, $P < 0.05$.

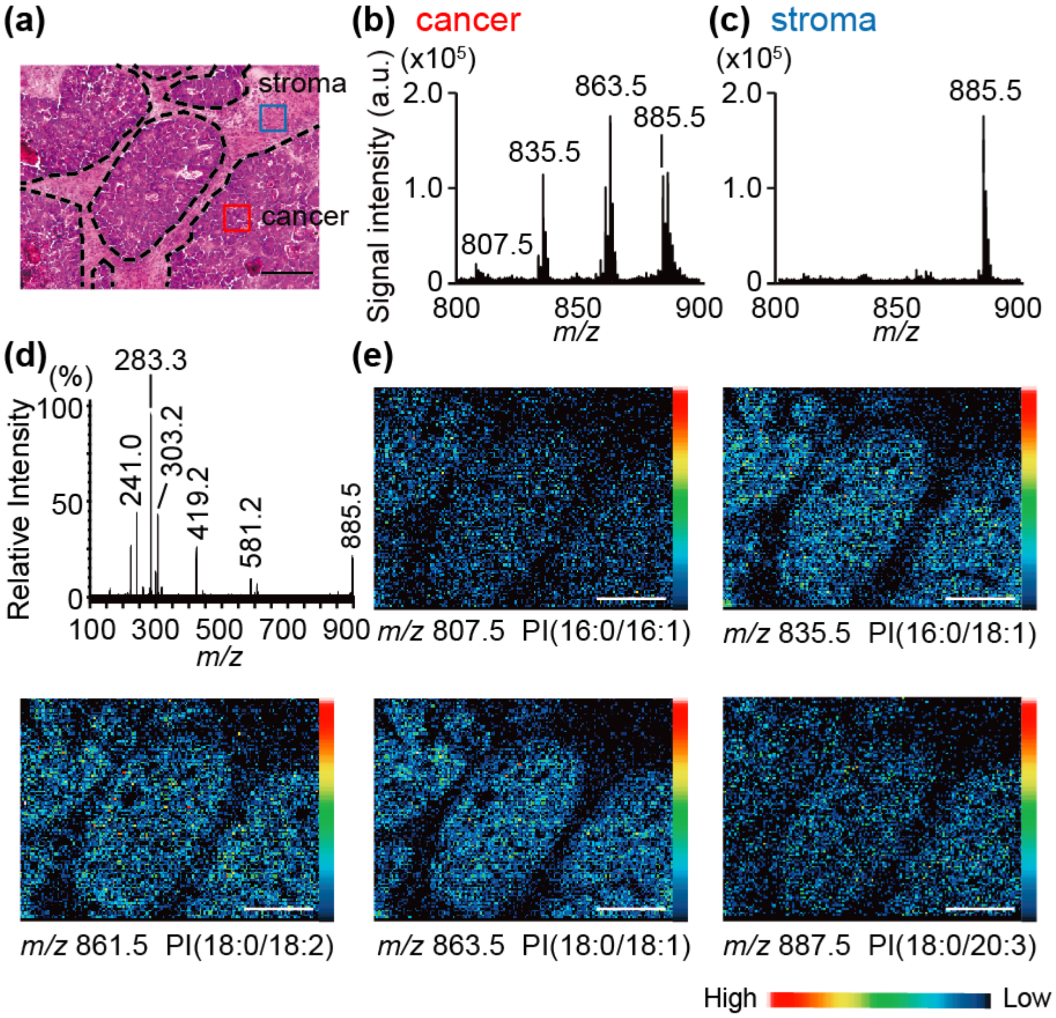

Supplementary Fig. S5. MALDI-IMS of colorectal cancer tissues. (a) A representative photomicrograph of HE staining of colorectal cancer tissues. The dotted lines indicate the borders between cancer cells and stromal regions. Scale bar, 200 μm. Boxed areas red and blue were regions used to acquire averaged mass spectra. **(b)** and **(c)** Averaged mass spectra ranging from *m/z* 800 to 900 obtained from cancer cells (b) and stroma (c). **(d)** Tandem mass spectra of product ions derived from *m/z* 885.5 in colorectal cancer cells. **(e)** PI(18:0/20:4) MALDI-IMS images of the indicated molecules with the indicated *m/z* values.
